# Almond Allergy in Children and Adults: A Narrative Review of Current Knowledge, Clinical Challenges, and Research Gaps

**DOI:** 10.3390/nu18050831

**Published:** 2026-03-04

**Authors:** Tudini Laura, Colletti Giorgio, Iavarone Sonia, Moraca Paola, Brindisi Giulia, Zicari Anna Maria, Anania Caterina

**Affiliations:** Department of Maternal, Infantile and Urological Sciences, La Sapienza University of Rome, 00161 Rome, Italysonia.iavarone@uniroma1.it (I.S.); paola.moraca@uniroma1.it (M.P.); giulia.brindisi@uniroma1.it (B.G.); annamaria.zicari@uniroma1.it (Z.A.M.); caterina.anania@uniroma1.it (A.C.)

**Keywords:** almond allergy, tree nut allergy, oral food challenge, component-resolved diagnostics, food allergy

## Abstract

**Background:** Almond is one of the most widely consumed tree nuts worldwide; however, almond allergy remains poorly characterized. Despite frequent sensitization, the prevalence of clinically relevant almond allergy appears low, contributing to diagnostic uncertainty. This review summarizes current evidence on the epidemiology, clinical manifestations, and diagnostic challenges of almond allergy. **Methods:** A narrative review was conducted using PubMed, Scopus, and UpToDate databases. Studies reporting almond-specific data on epidemiology, diagnostics, molecular allergens, and oral food challenge (OFC) outcomes were included. **Results:** Across heterogeneous studies, clinically confirmed almond allergy appears to be uncommon despite high rates of sensitization, particularly among patients with atopic dermatitis and concomitant tree nut allergy. In sensitized individuals, OFC positivity ranges from 4% to 33%, with anaphylaxis and severe reactions reported in 0.5–12.2% of challenged patients. Conventional diagnostic tests, including skin prick testing and almond-specific IgE, demonstrate limited predictive value, with no reliable cut-off levels for predicting clinical reactivity. Consequently, OFC remains essential for definitive diagnosis. Clinical outcomes vary according to age, ethnicity, and almond processing, with lower OFC positivity observed in pediatric cohorts and reduced reactivity to processed almond products. **Conclusions:** Almond allergy is relatively rare despite frequent sensitization. Improved almond-specific molecular diagnostics may enhance risk stratification and reduce unnecessary dietary avoidance.

## 1. Introduction

Food allergy (FA) is an expanding Western public health challenge with an estimated prevalence of up to 10% of the general population (8% of children) [1,2,3].

The so-called “Big Eight” food allergens (cow’s milk, egg, wheat, soy, peanut, tree nuts, fish and shellfish) account for the majority of FA cases with a significant higher frequency in children than in adults [4,5,6].

Within this group, tree nut allergies (TNAs) are generally more severe and potentially life-threatening compared to milk or egg allergies, which are typically mild and transient [4,7,8]. Among tree nuts, almonds are one of the most consumed ones worldwide. With a kernel output of approximately 1.2 million tons, almond rank first among the world’s four major dry nuts [9,10].

From an epidemiological standpoint, the accuracy of prevalence data for individual tree-nut allergies is limited, as tree nuts comprise heterogeneous species rather than a single, uniform group. Their prevalence varies substantially according to age and geographic region. Although some studies report relatively high rates of almond sensitization or self-reported allergy (13.30%) [11], current evidence does not robustly support the claim that almond allergy is the fourth most common tree-nut allergy.

The almond (*Prunus dulcis*) belongs to the Rosaceae family and is native to Southwest Asia. It is part of the subfamily Amygdaloideae, which also includes apricots (*Prunus armeniaca*), cherries (*Prunus avium*), nectarines (*Prunus persica*), peaches (*Prunus persica*), and plums (*Prunus domestica*).

The nutritional properties of almonds have been extensively documented. They are rich in mono- and polyunsaturated fatty acids and contain significant levels of phytosterol and lipophilic steroid alcohol, which inhibit cholesterol reabsorption. Almonds are also an excellent source of vitamin E tocopherols (α, β, γ, δ) and riboflavin (vitamin B2) [9].

The high content of these micronutrients contributes to the well-recognized health benefits associated with almond consumption, including a reduced risk of cardiovascular disease and diabetes as well as enhanced antioxidant activity [12,13,14,15].

In this paper, we aim to consolidate the existing literature on almond allergy, providing a comprehensive overview of its clinical presentations, diagnostic strategies and current gaps in knowledge.

### 1.1. Pathophysiology

Almond allergy is triggered by an abnormal response of the immune system to specific proteins found in almonds, which are mistakenly recognized as harmful. This response is primary mediated by immunoglobulin E (IgE), which plays a central role in activating immune cells such as mast cells and basophils [16].

Less commonly, FA is caused by both IgE- and non-IgE-mediated reactions; an example is mucosal inflammation in patients with eosinophilic esophagitis [17]. Clinical manifestations of FA depend not only on the type of immune response and mediators released, but also on the tissue location where these mediators act [16].

To date, two main forms of almond allergy are known: primary almond allergy, caused by sensitization to the legumin Pru du 6, which can clinically manifest with moderate to severe symptoms; and a secondary, milder form, occurring in birch-endemic areas, due to cross-reactivity resulting from structural homology between Pru d 1 and Bet v 1 [18]. As described in Table 1, other almond allergens, such as Pru du 8 (α-hairpinin) and Pru du 10 (mandelonitrile lyase), have been identified and may further refine molecular diagnostic profiles [19].

Pru du 6 is the most well-studied allergen among those derived from almonds. It is an 11S seed storage protein, representing approximately 60% of the total almond protein content [20]. Sensitization to the almond legumin Pru du 6 is highly specific for almond allergy and can therefore be considered a biomarker of symptomatic almond allergy [21].

Pru du 1, one of the main allergens involved in almond allergy, belongs to the PR-10 protein family, which is known for cross-reactivity with homologous proteins found in pollen and other plants of the Rosaceae family, such as apples and peaches [18]. This molecule is involved in allergic reactions, particularly in patients with birch pollen allergy. Pru du 3 is a non-specific lipid transfer protein 1 (NsLTP1), a class of proteins involved in plant defense against pathogens and other stress factors [22]. Pru du 3 shows immunological similarity with LTPs from other plant foods. Due this cross-reactivity, individuals sensitized to Prup d 3 may also react to other LTP-containing foods [23]. To date, there are no references regarding the allergenicity of Pru du 3, and studies on this almond allergen remain very limited [19]. Pru du 4 is a profilin, which is considered a panallergen. Profilins were first identified as birch pollen allergens and were later recognized as food allergens present in fruits, vegetables, and nuts [24]. Although less common in almond allergy, Pru d 4 can bind IgE, particularly in pollen-sensitized individuals, and is typically associated with mild reactions, often limited to the oral cavity. Minor allergens have also been described, such as vicilins, which may be involved in the immune response in individuals sensitized to multiple types of nuts and seeds [25]. In addition, other almond allergens have been described in the literature, including Pru du 2, a thaumatin-like protein that is stable to heat, pH variation, and proteolysis [26] and shows very high sequence identity with the homologous peach allergen Pru p 2 [27]. Pru du 5, moreover, has been identified as a 60S acidic ribosomal protein and it exhibits 81% sequence identity and 94% homology with the tomato allergen ARP60S [28]. These findings indicate limited allergen specificity and suggest significant cross-reactivity in both cases.

### 1.2. Clinical Manifestations

Almonds are known to contain several allergenic proteins that can induce a wide spectrum of clinical reactions, ranging from mild symptoms to life-threatening anaphylaxis. The nature and severity of these manifestations are often influenced by factors such as the degree of sensitization, the type of allergenic protein involved, and the amount of allergen ingested [29].

Oral Allergy Syndrome (OAS) is one of the most common clinical manifestations of almond allergy, particularly in individuals with birch pollen allergy and it is defined as the symptoms of IgE-mediated immediate allergy localized in the oral mucosa. As demonstrated by Kabasser et al. in 2022 [18], subjects sensitized to birch pollen frequently exhibit cross-reactivity to almond proteins, especially Pru du 1, a Bet v 1-homologue. This protein, along with others like Pru du 6, can induce itching, swelling or tingling in the oral cavity, especially after eating consumption of raw almonds or almonds present in processed foods. These symptoms usually occur shortly after exposure and are often self-limiting. Cutaneous reactions are also common in patients with almond allergy, ranging from mild urticaria to more extensive eczema or angioedema. According to Bezerra et al. (2021) [30], skin reactions may be triggered not only by ingestion, but also by contact with almond-containing products. This finding highlights the potential for allergic reactions following skin exposure to almond residues, particularly in food handling or cooking environments. Gastrointestinal symptoms, which are more frequently observed in children, can vary in severity depending on the amount of almond consumed and the individual’s level of sensitivity [29]. These symptoms may include nausea, vomiting, abdominal pain and diarrhea. These symptoms generally occur within a few minutes of almond ingestion and are often indicative of a type I hypersensitivity reaction. Respiratory symptoms may be more severe and involve the upper and lower respiratory tract. Mild forms include nasal congestion, sneezing, and itching of the throat and eyes, indicating a more localized reaction. Moderate-to-severe respiratory symptoms such as wheezing, cough, and shortness of breath are often indicative of an IgE-mediated allergic response and tend to be more frequent in individuals with pre-existing asthma or other atopic conditions, underscoring the complex interplay involved in nut allergy [31]. Anaphylaxis represents the most serious and potentially life-threatening manifestation of almond allergy. IgE-mediated anaphylaxis to almond is an acute, systemic allergic reaction triggered by almond exposure in sensitized individuals and may include hives, angioedema, laryngeal edema, difficulty breathing, abdominal pain, hypotension and loss of consciousness. The risk of anaphylaxis is particularly high in patients who have a history of severe allergic reactions to other nuts or in individuals with a family history of anaphylaxis [32]. Additionally, the presence of cross-reactivity with other allergens, such as birch pollen or other nuts, may increase the likelihood of a severe reaction in sensitized subjects [33]. Moreover, a small portion of patients may experience anaphylaxis triggered by physical exercise, following almond. This condition known as Food-Dependent Exercise-Induced Anaphylaxis (FDEIA) is relatively rare, but, it can be fatal if not promptly recognized and treated [34].

### 1.3. Diagnosis

Almond allergy represents an unusual and paradoxical issue in clinical allergy. While population-based studies suggest that up to 2% of individuals show almond sensitization with detectable almond-specific IgE antibodies and/or positive skin prick tests (SPT), fewer than 0.1% actually develop clinical symptoms when assessed through controlled oral food challenges (OFC) [31,35,36]. This marked discrepancy between sensitization and true allergic reactivity poses important challenges in both diagnosis and patient management. At a molecular level, almond allergy is driven by a heterogeneous set of allergenic proteins, each characterized by distinct biochemical properties and clinical implications, as previously described. 

While component resolved diagnosis (CRD) has significantly enhanced the precision of FA diagnosis by enabling the identification of IgE responses to specific almond proteins, it is not without limitations. Traditional diagnostic tests, such as SPTs and whole extract-specific IgE assays, offer high sensitivity (up to 94%) but relatively poor specificity (as low as 33%) [21]. CRD improves diagnostic accuracy by identifying risk-associated molecular profiles, such as positivity to Pru du 6 or Pru du 3, thereby increasing the pretest probability of true clinical allergy. In contrast, isolated sensitization to Pru du 1 or Pru du 4 suggests benign cross-reactivity and may not warrant strict avoidance of almond [21].

Nevertheless, the current availability of molecular testing tools remains imperfect. Widely used multiplex platforms such as ImmunoCAP ISAC^®^ 112 and ALEX^®^ v2 do not include Pru du 6, Pru du 8, or Pru du 10, which are among the most clinically predictive components [37]. This gap requires clinicians to interpret negative multiplex results with caution and often necessitates the use of additional singleplex assays or referral to specialized reference laboratories, resulting in increased costs and diagnostic delays [30]. A unified multiplex panel that includes all major almond components would significantly improve access to precise molecular diagnostics and help reduce the overuse of oral food challenges [37].

Despite these advances, OFC remains the gold standard for the diagnosis of almond allergy. Under controlled medical supervision, incremental doses of almond protein are administered during the challenge, with some protocols using a ‘top dose’ of approximately 3 g, while others have used doses of up to 6 g. The challenge is continued until objective symptoms occur or clinical tolerance (or sustained tolerance) is confirmed [37]. Across multiple cohorts, including studies by Baker & Kattan and Virkud et al. OFCs report pass rates consistently exceeding 90%, with anaphylaxis observed in less than 1% of cases [35,36]. These findings indicate that the majority of sensitized patients are not clinically allergic and can safely reintroduce almond into their diets following a negative challenge.

Almond allergy exemplifies the complexity of FA diagnosis in the era of molecular medicine. While conventional tests remain valuable for initial screening, the integration of CRD with oral challenge allows for more accurate risk stratification. The presence of specific IgE to high-risk components like Pru du 6 or Pru du 3 informs clinical decision-making, whereas recognition of benign sensitization patterns may prevent unnecessary dietary avoidance.

### 1.4. Prevention and Therapy

The prevention and treatment of almond allergy, and more broadly, tree nut allergies, represent a dynamic field of research with evolving clinical recommendations.

Historically, strategies for the prevention of food allergies, particularly those involving highly allergenic foods such as nuts, were based on delaying their introduction into the infant diet [38,39]. Over the past decade, however, this paradigm has shifted fundamentally, driven by robust evidence indicating that early and regular exposure to food allergens may, paradoxically, reduce the risk of subsequent allergy development [40].

A crucial feature of current preventive strategies is the concept of oral tolerance, which refers to the immune system’s ability to recognize a food antigen as harmless, thereby preventing an allergic reaction. This understanding has been profoundly informed by landmark studies such as the Learning Early About Peanut Allergy (LEAP) trial [41]. Although the LEAP study focused specifically on peanut allergy, it demonstrated a marked reduction in allergy incidence among high-risk infants who were introduced to peanut between 4 and 11 months of age and who maintained regular consumption. The immunological mechanism underlying these findings, namely the induction of oral tolerance, is considered broadly applicable to other major food allergens, including tree nuts.

Consequently, leading scientific societies, such as the European Academy of Allergy and Clinical Immunology (EAACI), have revised their guidelines. The 2020 EAACI guidelines recommend introducing complementary foods, including potential allergens, between 4 and 6 months of age, while explicitly advising against introduction before 4 months [40]. 

Although data specifically addressing early almond introduction remain limited, evidence from broader studies on tree nuts and multiple allergenic foods is informative. The PreventADALL trial, which evaluated early introduction of several allergenic foods, demonstrated that regular exposure from as early as 3 months of age significantly reduced the risk of documented FA at 36 months [42]. Consistently, a systematic review and meta-analysis reported that earlier introduction of multiple allergenic foods, including tree nuts such as almond, was associated with a reduced risk of IgE-mediated FA [43]. Nonetheless, implementation challenges remain. The PreventADALL study reported lower-than-expected adherence rates, particularly in groups undergoing combined interventions. Moreover, the optimal minimum dose and frequency of exposure required for effective allergy prevention, including for almond, have yet to be clearly defined [42].

Beyond prevention, the therapeutic management of established almond allergy is also evolving. Oral immunotherapy (OIT) has emerged as a promising therapeutic strategy aiming to desensitize allergic individuals and increase their threshold for allergic reactions. The goal of OIT is to allow patients to tolerate accidental ingestion of the allergen or, in some cases, to regularly consume small quantities of the previously avoided food. Studies have demonstrated the efficacy and safety of OIT for FA in adults [44] and for multifood OIT in children aged 1 to 18 years in academic pediatric clinics [45]. Specifically for tree nut allergy, real-world safety analyses of preschool OIT have also shown encouraging results [46]. Evidence from studies of tree nut OIT, including almond, suggest that this approach is feasible and practicable. A systematic review of tree nut allergy management identified oral immunotherapy, administered as single or multi-nut protocols with or without Omalizumab, as the most extensively studied intervention and found it effective in reducing the risk associated with accidental exposures [32]. A multicenter, real-world safety analysis of preschool tree nut OIT including almond reported that 96.7% of patients achieved a maintenance dose. During the buildup phase, 70.7% experienced reactions, predominantly mild, and only 2.17% required epinephrine. Among 92 patients initiating tree nut OIT, 85.9% underwent single-food therapy, which appeared safe and well tolerated [46]. To support standardized OIT protocols, irradiated tree nut flours for almond, walnut, cashew, and hazelnut have been developed, meeting FDA standards for microbial safety while maintaining stable protein and allergen content over a 24-month period [47]. Collectively, these findings support the feasibility and relative safety of almond OIT, particularly when initiated in preschool-aged children, provided that appropriate patient selection and monitoring are ensured [46,48].

Despite its potential, OIT is not without risks. Reports highlight the possibility of severe anaphylactic reactions to home doses of OIT [49], underscoring the critical need for careful patient selection, expert medical supervision, and comprehensive patient education throughout the treatment process.

Furthermore, novel therapeutic avenues are being actively explored. Omalizumab, a monoclonal antibody targeting IgE, has shown promise in the treatment of multiple food allergies [50], potentially paving the way for desensitization strategies, including almond allergy.

Although almond-specific data remain limited, the OUtMATCH trial, systematic review and subsequent systematic reviews and meta-analysis demonstrated that Omalizumab treatment significantly increased reaction thresholds to peanut and other common food allergens in individuals with multiple food allergies [51,52,53]. Moreover, when combined with OIT, Omalizumab significantly improves desensitization rates and tolerated doses compared with placebo plus OIT [52,53].

The 2024 FDA approval of Omalizumab for the treatment of single or multiple food allergies in individuals aged 1 year and older represents a major therapeutic advance, although its primary benefit lies in protection against accidental exposure rather than enabling unrestricted consumption of allergenic foods [54,55].

Finally, it is important to acknowledge that percutaneous sensitization may contribute to the development of food allergies. A study investigating percutaneous sensitization to almond oil indicated that topical exposure may play a role in allergy development [56].

Research on gourmet nut oils processed using different methods demonstrated that almond oil may retain residual allergenic proteins depending on the degree of processing, with less intensively processed oils showing higher protein concentrations and greater IgE-binding capacity [57]. The main almond allergens may remain immunologically active in oil extracts, particularly when subjected to minimal processing [58]. The presence of allergenic proteins in almond-containing topical products, together with broader evidence linking impaired skin barrier function to food sensitization, highlights the importance of considering cutaneous exposure pathways in allergy pathogenesis [29,30].

The evolving understanding of almond allergy, from early introduction for prevention to advanced desensitization and adjunctive therapies, represent a significant step forward in improving outcomes for affected individuals.

## 2. Materials and Methods

In this narrative review, a comprehensive literature search was conducted using MEDLINE via PubMed, UpToDate and Scopus dashboards. The following keywords were applied: “almond allergy, tree nut allergy, almond challenge, almond antigens”. All original studies were considered, including translational investigations, case reports, observational studies, retrospective and prospective clinical trials. The search for “almond allergy” retrieved a total of 147 records, of which 36 were identified as translational studies and 105 were deemed not pertinent to the topic. Based on predefined inclusion criteria, six studies were ultimately included. The search using the term “tree nut allergy” retrieved 576 records, 567 of which did not provide almond-specific information; consequently, nine studies were included from this search. Overall, a total of 15 studies were selected for the present analysis across both search strategies. The selected manuscripts were critically examined, and the main findings are described in the text and summarized in the tables. Despite the limited number of studies specifically addressing almond allergy, and for the sake of consistency, studies focusing on nut allergies in general were excluded.

## 3. Clinical Studies

**Figure 1:**
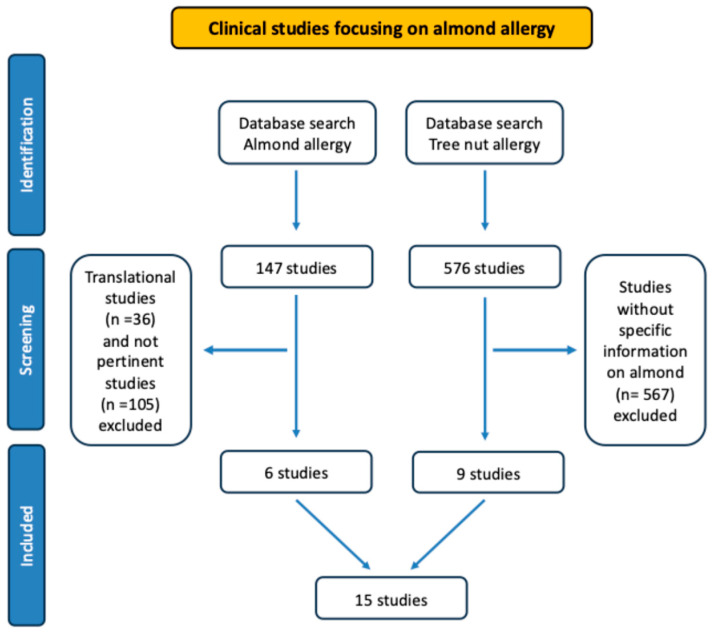
PRISMA Figure Flow Diagram.

In Table 2 and Table 3 and Figure 1 are listed the main studies addressing epidemiology of almond allergy and OFC outcomes.

**Figure 2:**
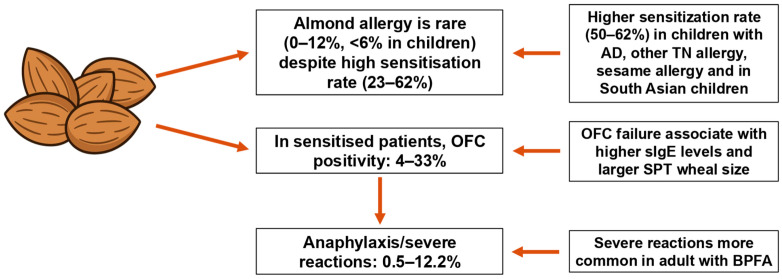
Epidemiology, diagnosis, and clinical severity of almond allergy. AD: Atopic Dermatitis; TN: tree nuts; OFC: Oral Food Challenge; sIgE: serum IgE; SPT: Skin Prick Test; BPFA: birch pollen-related food allergy.

As shown in Figure 2, almond allergy is characterized by a marked discrepancy between high rates of sensitization and a low prevalence of confirmed clinical allergy. In sensitized individuals, OFC positivity remains limited, although severe reactions, including anaphylaxis, may occur in selected populations. Diagnostic failure is associated with higher almond-specific IgE levels and larger SPT wheal diameters. Clinical outcomes are influenced by age, ethnicity and underlying birch pollen-related food allergy (BPFA).

## 4. Discussion

The available literature on almond allergy, although heterogeneous, consistently suggests that true clinical allergy to almond is relatively uncommon despite high rates of sensitization. Large retrospective studies, such as those by Virkud et al. [35] and Baker et al. [36], report OFC positivity rates of only 4–5%, with anaphylaxis occurring in approximately 0.5% of challenged patients. These findings are further supported by prospective data from Elizur et al. [59], in which only 2% of sensitized individuals were confirmed allergic by OFC, reinforcing the notion that almond sensitization frequently reflects clinical tolerance rather than true allergy.

Several studies highlight the limited predictive value of conventional diagnostic tools. Almond-specific IgE levels and SPT wheal diameters correlate with OFC outcomes at a population level; however, no reliable cut-off achieving a 95% positive predictive value has been identified. This was clearly demonstrated by Ho et al. [60], who showed that even large wheal diameters yielded modest likelihood ratios, and by Elizur et al. [59], who found that neither SPT nor BAT cut-offs were capable of definitively predicting clinical reactivity. These findings underscore the continued reliance on OFCs for definitive diagnosis.

Importantly, population characteristics strongly influence reported outcomes. Pediatric cohorts consistently show lower rates of confirmed almond allergy compared to adults. This discrepancy is exemplified by the study of Kallen et al. [58], the only investigation conducted exclusively in an adult population, which reported a markedly higher OFC positivity rate (33.3%). In contrast, pediatric and mixed cohorts generally report positivity rates of approximately 5%. Ethnic differences may also play a role, as demonstrated by Luyt et al. [62], who reported significantly higher rates of both sensitization and clinical allergy among South Asian children compared to White children. The ethnicity reflects geographical origin and is associated with environmental and dietary exposures rather than genetic predisposition.

A critical and underexplored variable is almond processing. The study by Kallen et al. [58] provides compelling evidence that processing profoundly affects clinical outcomes: all OFCs performed with processed almonds were negative, whereas one-third of challenges with raw almonds were positive, including severe reactions. This observation aligns with the high prevalence of birch pollen sensitization in that cohort and supports the hypothesis that thermal processing reduces the allergenicity of PR-10 proteins in almond. The lack of specification regarding almond form (raw vs. processed) in most previous studies represents a significant methodological limitation and likely contributes to the variability observed across published results.

Beyond PR-10-mediated allergy, rare but severe phenotypes have been described. Case reports by Senders et al. [69] and Betancor et al. [34] document almond-induced food-dependent exercise-induced anaphylaxis (FDEIA), including the identification of a novel almond allergen belonging to the 7S globulin (vicilin) family. These reports underscore that, although uncommon, almond allergy may involve non-PR-10 proteins and can present with life-threatening reactions.

Finally, several studies suggest that co-sensitization does not necessarily imply co-allergy. Almond sensitization frequently occurs in patients allergic to peanut, sesame, or other tree nuts; however, confirmed almond allergy remains rare. Couch et al. [61] and Elizur et al. [59] both suggest that almond may often be safely introduced into the diet of sensitized individuals, potentially without extensive pre-testing, although this approach requires careful patient selection.

## 5. Conclusions

This review highlights the current paucity of scientific literature specifically addressing almond allergy, which remains a frequently under-recognized and under-reported condition within the broader landscape of food allergies. Despite increasing awareness of nut-related allergies overall, almond allergy continues to be relatively neglected in both clinical practice and research, resulting in significant knowledge gaps regarding its true prevalence, clinical spectrum, and natural history.

The available evidence suggests that almond sensitization is common, whereas confirmed clinical allergy is comparatively rare, and that currently available diagnostic tools, including SPT, sIgE and BAT, have limited predictive value. Consequently, OFC remain essential for diagnosis, despite its inherent risk and practical limitation. Emerging data further indicate that factors such as age, ethnicity, and, critically, almond processing may substantially influence clinical reactivity, underscoring the need for greater standardization in diagnostic approaches and study methodologies.

Looking ahead, advances in molecular allergology are expected to facilitate the identification and characterization of clinically relevant almond allergens, paving the way for the development of more accurate and reliable, almond-specific diagnostic tools. Such progress would enable a more precise distinction between clinically relevant allergy and asymptomatic sensitization, while also supporting a deeper understanding of the heterogeneous clinical phenotypes associated with almond allergy.

## Figures and Tables

**Table 1:** Major almond allergen components and their main characteristics.

Allergen	Family	Stability	Clinical Signal
Pru du 1	PR-10	Labile	Birch-pollen OAS
Pru du 2	TLP (PR-5)	Very stable	Potential cross-reactivity among Rosaceae
Pru du 3	nsLTP	Very stable	LTP syndrome; severe systemic reactions
Pru du 4	Profillin	Labile	Mild OAS; pan-allergen
Pru du 5	60sRP	Likely labile (no direct data available)	Potential cross-reactivity with tomato
Pru du 6	11S legumin	Very stable	Systemic allergy marker
Pru du 8	Hairpinin	Stable	Highly specific; confirmatory marker
Pru du 10	MDL	Stable	Emerging marker; research stage

OAS: Oral allergy syndrome; TLP: Thaumatin-like protein; LTP: Lipid transfer protein.

**Table 2:** Main studies focusing on almond allergy.

Author, Year (Country)	Study Design (*n*)	Study Population	Diagnostic Method	Main Results	Key Findings
Virkud et al., 2019 (USA) [35]	Retrospective (590)	1–66 yrs; almond SPT and sIgE positive	OFC	OFC negative: 92%; OFC positive: 5%; indeterminate: 3%; anaphylaxis: 0.5%	OFC failure associated with higher almond sIgE and larger SPT wheal size
Baker et al., 2018 (USA) [36]	Retrospective (400)	0.5–25 yrs; almond SPT and sIgE positive	OFC	OFC negative: 94%; OFC positive: 4%; indeterminate: 2%; anaphylaxis: 0.5%	Higher sIgE and SPT wheal size correlated with OFC failure
Kallen et al., 2025 (Netherlands) [58]	Retrospective (94)	Adults (>18 yrs); almond SPT and sIgE positive	OFC (raw vs. processed almond)	Raw almond OFC: 33.3% positive, 12.2% anaphylaxis; processed almond OFC: 100% negative	Almond processing strongly influences allergenicity; PR-10-mediated reactions prominent in birch pollen-sensitized adults
Elizur et al., 2018 (Israel) [59]	Prospective cohort (83)	>3 yrs; suspected IgE-mediated TN allergy	OFC, SPT, sIgE, BAT	Almond sensitization: 59%; confirmed allergy: 2%	Almond allergy is rare; BAT predicts tolerance; no SPT or BAT cut-off achieved 95% PPV
Ho et al., 2006 (Australia/China) [60]	Retrospective (680)	0.3–19 yrs; TN and/or sesame evaluation	SPT, OFC	No SPT wheal diameter reached 95% PPV (LR max 4.17)	SPT wheal size insufficient to reliably predict almond OFC outcome
Couch et al., 2017 (USA) [61]	Retrospective (109)	4–19 yrs; TN-allergic patients	OFC	Almond sensitization: 61%; OFC positive: 0%	Almond may be safely introduced in many peanut-allergic patients
Luyt et al., 2016 (UK) [62]	Prospective (2638)	Pediatric referrals	SPT, clinical history	Almond sensitization: South Asian 61.9%, White 31.1%; allergy: 7.4% vs. 1.8%	Significant ethnic differences in sensitization and clinical allergy
Boyano et al., 2015, Spain [63]	Prospective (96)	3–18 months without TN allergy	SPT, sIgE	Almond sensitization: 26.2% Higher sensitization rate in children with AD	Almond sensitization is common in infancy and is associated with environmental exposure and AD
Anagnostou, 2019, USA [64]	Prospective (78)	3–17 yrs peanut allergic	SPT	Almond sensitization: 38% (median SPT wheal 4 mm). Almond/hazelnut co-sensitization: 24%.	High almond sensitization in peanut-allergic children, with frequent hazelnut co-sensitization; consumption common regardless of SPT result
Ludman et al., 2013, Switzerland [65]	Retrospective (80)	3–16 yrs undergoing peanut and/or TN OFC; sensitized without prior consumption	OFC	14 almond OFCs: 4 positive, 10 negative	Limited data specifically addressing almond; majority of almond OFCs were negative
Sokol e al., 2020, USA [66]	Prospective (119)	4–14 yrs with positive sesame sIgE and reaction to ≥1 other food	sIgE, clinical history	Almond sensitization: 23%; almond allergy: 5.9%. 100% sesame-allergic patients co-sensitized to almond; 13.3% allergic to both. Median almond sIgE: 2.1 kUA/L overall; 4.4 kUA/L in sesame-allergic	Almond sensitization frequently co-occurs in sesame-allergic children; clinical co-allergy less common
Fleischer et al., 2005, USA [67]	Retrospective (278)	3–21.6 yrs with TN allergy	OFC	8–9% reaction to almond: 8 mild, 0 moderate, 1 severe reaction	Almond reactions infrequent and mostly mild. Patients allergic to ≥1 TN are unlikely to outgrow the allergy
Bird et al., 2012, USA [68]	Retrospective (16)	26–112 months with peanut allergy	sIgE	sIgE increased during the first 12 months of peanut OIT and decreased between 12–24 months	Peanut OIT did not significantly impact almond sensitization

**Table 3:** Case reports and rare phenotypes.

Author, Year (Country)	Study Type	Clinical Features	Key Findings
Senders et al., 2018 (Denmark) [69]	Case report	16-yr-old girl; PR-10 sensitization; FDEIA	First report of almond-induced FDEIA
Betancor et al., 2021 (Spain) [34]	Case report	Adult woman; severe almond-dependent FDEIA with rTTC	Identification of a novel almond 7S globulin (vicilin) allergen

Yrs: Years, SPT: Skin Prick Test, OFC: Oral Food Challenge, sIgE: serum IgE, TN: tree nuts, BAT: Basophil Activation Test, PPV: Positive predictive value, LRs: Likelihood ratios, AD: Atopic Dermatitis, OIT: Oral Immunotherapy, FDEIA: Food-dependent exercise-induced anaphylaxis, rTTC: Reverse Takotsubo Cardiomyopathy.

## Data Availability

The authors declare that the data supporting the findings of this study are available within the paper.

## Abbreviations

The following abbreviations are used in this manuscript:

OAS	Oral allergy syndrome
TLP	Thaumatin-like protein
LTP	Lipid transfer protein
YRS	Years
SPT	Skin Prick Test
OFC	Oral Food Challenge
sIgE	Serum IgE
TN	Tree nuts
BAT	Basophil activation test
PPV	Positive predictive value
LRs	Likelihood ratios
FDEIA	Food-dependent exercise-induced anaphylaxis
rTTC	Reverse Takotsubo Cardiomyopathy
AD	Atopic dermatitis
BPFA	Birch pollen-related food allergy
